# Global Basic Reproduction Number of African Swine Fever in Wild Boar and a Mental Model to Explore the Disease Dynamics

**DOI:** 10.1155/2024/1046866

**Published:** 2024-03-09

**Authors:** Shraddha Tiwari, Thakur Dhakal, Tae-Su Kim, Seong-Hyeon Kim, Sang-Joon Lee, Dae-Sung Yoo, Ho-Seong Cho, Gab-Sue Jang, Yeonsu Oh

**Affiliations:** ^1^Department of Life Science, Yeungnam University, Gyeongsan 38541, Republic of Korea; ^2^College of Veterinary Medicine and Institution of Veterinary Science, Kangwon National University, Chuncheon 24341, Republic of Korea; ^3^College of Veterinary Medicine, Chonnam National University, Gwangju 61186, Republic of Korea; ^4^College of Veterinary Medicine and Bio-Safety Research Institute, Jeonbuk National University, Iksan 54596, Republic of Korea

## Abstract

Basic reproduction number (*R*_0_) is a mathematical expression used in epidemiology to address expected number of secondary cases. *R*_0_ helps to predict outbreak diffusion and preventive measures. As African swine fever (ASF) is a viral infectious disease, there are significant studies related to *R*_0_ of the ASF outbreak, but most of them are investigated in a zonal and geospecific boundary. This study explores the general overview of African swine fever virus (ASFV) *R*_0_ based on existing literature and examines for the global scale using a doubling time approach using wild boar outbreaks. Further, a qualitative mental model is developed to explore the ASF disease dynamics. It was found that the average *R*_0_ was 3.56 from existing literature. Based on the global scenario, ASFV spread in wild boar was above the threshold line (*R*_0_ ≥ 1). The recent growth trend (*R*_0_=5.87) flagging the very high risk. ASFV is threatening the world. The qualitative mental model highlighted the veterinary services as awareness and R&D support are highlighted as the control measures. This study provides a reference to researchers and veterinarians in setting strategies of developing preventive measures and highlights the importance of awareness programs and R&D support for mitigating the ASFV spread.

## 1. Introduction

The disease spread and disease dynamics parameters such as Reproduction number (*R*_o_), incidence rate, prevalence rate, serial interval, and contact rates are examined and reported frequently for sustainable preventive practice. *R*_0_, also known as basic reproduction ratio, measures a disease's ability to spread in a population. It is an average number of secondary cases caused by an infectious individual during the infectious period (early stage of an outbreak). The incidence rate measures the number of new cases over a specific period. The prevalence rate explores the population of individuals in a population who were infected by a disease at a specific time. Serial interval presents the time between the onset of symptoms in primary and secondary cases, and the contact rate gives information about the contacts per unit of time between susceptible and infected individuals [1, 2]. Without considering other measures, the research focuses on the *R*_0_ of African swine fever virus (ASFV) in wild boars for the global scenario.

African swine fever (ASF) has spread in many countries since it was first reported in Kenya in 1921 [3]. ASFV was eradicated from all infected countries in the mid-1990s. The reintroduction of ASFV in Georgia in the Caucasus was reported in 2007, then again spreading worldwide [4]. Since then, there have been significant studies investigating the dynamic parameters and developing strategies to mitigate the adverse impact of ASFV [5]. Wild pigs and the pig industry are at risk due to ASFV. The virulence of ASFV isolates varies, ranging from highly virulent isolates that can kill up to 100% of swine to moderately or low virulent isolates that can cause mortality rates between 3% and 100% [6, 7].

Wild boars roam freely in the forest, and it is difficult to examine the exact population [8]. Wild boar is considered the significant driver of the spread of ASFV, though they become sick and die. When ASFV is identified on a domestic farm, mass culling is adopted and applied as a strict control measure which is hard to follow in the case of wild boars. The ASFV spread parameters in wild boars reveal the recent disease scenario and support for setting mitigating measures. Understanding disease growth in global scenario is crucial for the effective implementation of one health policies [9]. Our literature survey found that *R*_0_ of ASFV was examined based on geospatially focused area but not in the global contest. Therefore, this research aims to collect and summarize the information from the literature on *R*_0_ of ASF, examine *R*_0_ for wild boars in the global scenario, and offer insight into epidemic control measures highlighting through mental model.

## 2. Materials and Methods

### 2.1. Data

**Figure 1:**
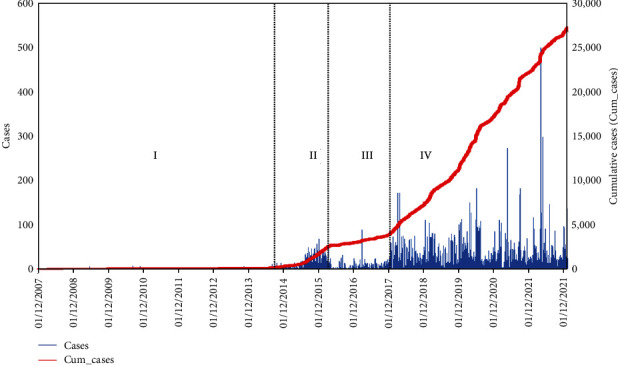
ASF outbreaks in wild boars (source: [10]).

Two approaches were adopted to analyze the *R*_0_ of ASF. First, a literature search was conducted on *R*_0_ for different regions and time frames. A total of 19 research about the *R*_0_ of ASF published in different time frames and geographic regions were examined, obtained from the Google Scholar database. The records of *R*_0_ for ASF were tabulated, and the average of them was extracted to get general information for global scenarios. Second, the global surveillance data reported in FAO data portal [10] were extracted and *R*_0_ for wild boar was calculated using a doubling time approach [11]. The recorded ASF cases and cumulative cases for wild boar from 1 December 2007 to 30 December 2022 can be seen in Figure 1. Visualization of the growth curve, the entire survey period was divided into four phases: I (2,224 days) between 01/12/2007 and 01/01/2014, II (814 days) between 02/01/2014 and 25/03/2016, III (627 days) between 26/03/2016 and 12/12/2017, and final phase IV (1,851 days) between 01/12/2007 and 30/12/2022.

### 2.2. *R*_0_ from Doubling Time Approach

Among various approaches for analyzing *R*_0_ in epidemiology, doubling time is the fast, easy to understandable, and most broadly applied method. The recent expansion of ASF worldwide is after its reintroduction in Europe in 2007. Therefore, this study examines the data recorded in the FAO epidemiology data portal empress-i between 1 December 2007 and 30 December 2022. It is considered that the disease outbreaks were growing at an exponential rate following Equation (1).

(1) $$\begin{array}{c} y_{\text{t}}=y_{0}e^{\lambda t}, \end{array}$$

where *y*_t_ is an outbreak at time *t*, *y*_0_ is the initial outbreak, and *λ* is the growth rate. The average doubling time (*T*_d_)of outbreaks is the time when the outbreak is double the initial cases, which can be calculated as Equation (2).

(2) $$\begin{array}{c} T_{\text{d}}=\frac{\text{ln}\left(2\right)}{\lambda }∼\frac{0.70}{\lambda }. \end{array}$$

The symbol *R*_0_ ( = “R nought” or “R zero”) is widely accepted as a symbol for the basic reproduction number, sometimes called the even basic reproduction rate (even though it is not a rate) or basic reproductive number. *R*_0_ measures how secondary cases were generated in the early infected period. When an early period of infection, infectious period (*D*) was considered for disease growth, *R*_0_ can be mathematically expressed as Equation (3):

(3) $$\begin{array}{c} R_{0}=1+\frac{D}{T_{\text{d}}}\times \text{ln}\left(2\right). \end{array}$$

The *D* for ASF for wild boars was recorded with a varying range between 4 and 19 days [12]. Some other studies reported different *D*s for ASF [13] reported that the minimum infectious period ranged from 6 to 7 days and the maximum up to 40 days. Pershin et al. [14]: minimum of 4.1 days varying from 1 to 13, Lee et al. [15]: 3–19 days, Lim et al. [8]: 3.5 days. In this study, the *R*_0_ of ASF on a global scale was examined through Equation 3 using 6 days as the *D*, segmenting the cumulative cases through visualization into four phases of the observation period.

### 2.3. Mental Model of ASF Disease Dynamics

The mental model is a casual network of representations of beliefs on a complex system [16]. A mental model is a group of interconnected beliefs that affect how someone conceptualizes how the world works and anticipates the future [17]. A mental model was formulated based on the knowledge from literature and current understanding of ASF disease dynamics. A total of 23 variables related to disease spread and control measures were considered in the mental model. Variables such as climate change, contact rate, host population, and vector movements were considered drivers of disease spread.

## 3. Results

### 3.1. Literature Study

From the 19 literatures about the *R*_0_ of ASF both in domestic pig and wild boars, no identical values were obtained, varied with different geographic regions, study periods, and study methods (Table 1). Based on the surveyed literature, Belgium, China, Czech Republic, Georgia, Italy, Netherlands, Malta, Russia, South Korea, Uganda, Ukraine, and Vietnam are the countries where *R*_0_ was examined. The examination of secondary cases in recorded literature was evaluated with different approaches such as doubling time, network analysis and susceptible, infected, and removed (SIR) ratios. The highest *R*_0_ was recorded by de Carvalho Ferreira et al. [13] for Malta (18), and the minimum in the Netherlands (0.3) for domestic pigs reported by Eblé et al. [18]. When taking an average of reported values without considering host species, the overall *R*_0_ was 3.56. Similarly, the average infectious period from the record was 11.19 days, ranging from 2 to 39 days.

### 3.2. Estimation of *R*_0_ using Doubling Time

The *R*_0_ was estimated from the global ASF outbreaks in wild boars up to 30 December 2022 using a doubling time approach, considering the early phase of the disease and the *D* to be 6 days. It was found that the first phase had a low disease growth rate (0.03, *R*^*2*^ = 0.99) and, doubling time was 26.46 days followed, and phase IV; the recent phase between 13/12/2017 and 30/12/2022 had the highest growth rate (1.39, *R*^*2*^ = 0.99) and doubling time 0.50 days. The overall growth rate for the entire survey period was 0.37 per day, and the doubling time was 1.88 days. The *R*_0_ in phases I, II, III, III, and all phases together were 1.09, 2.52, 2.51, 5.87, and 2.29, respectively (Table 2). The increased outbreak growth rate and higher *R*_0_ in the recent phase flagged a greater threat of ASFV.

### 3.3. ASFV Dynamic Mental Model

**Figure 2:**
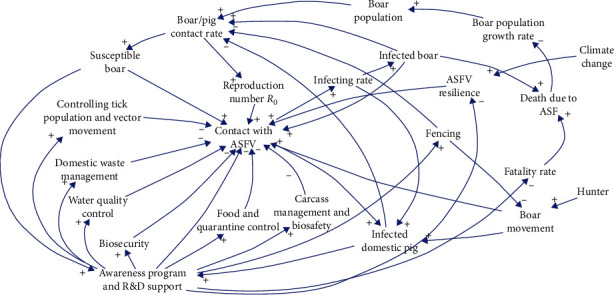
A mental model to overview the ASFV spread and control.

Disease spread dynamics is a complex system that can be simplified, and intelligent decisions can be made through mental models. Climate change influences the resilience of viruses [35]. The ASFV disease hosts are social animals, and their movement behavior and resilience power of virus in the environment directly impact the contact of ASFV. The mortality rate of ASFV is near 100% [36, 37], and death due to ASF decreases the boar population growth rate. Increasing the infecting rate of ASF, contact rate to disease host is increasing. Higher susceptible of boars and infected domestic pigs pressured to involve for R&D to develop vaccines and conduct awareness programs. Fencing is a measure to control the movement and minimize the contact rate of host species [38]. When the contact rate and infecting rate are increased *R*_0_ also increased. Controlling host movement, domestic waste management, water quality, biosecurity, carcass management and biosafety, and food and biosafety were assumed to be effective control measures in the proposed mental model. A total of 23 variables were used to develop a ASF spread, and control dynamics mental model visualized in a causal loop diagram (Figure 2). Active veterinary services [39] with awareness programs and R&D support were highlighted as major variables to control the spread. The model will help to understand how changes in one part of the system can affect other parts and identify potential leverage points for intervention or improvement.

## 4. Discussion

This study explored *R*_0_, one of ASF's epidemiological parameters, and developed a mental model to understand the spread and control. *R*_0_ projects the level of immunization that a community needs in order to develop immunity, control the spread of the infection, and be protected from contracting the disease [40]. The common interpretations about *R*_0_ are: if *R*_0_ < 1 disease is eradicated, that virus is almost dying out, the threshold point *R*_0_ = 1 the controlled, 1 < *R*_0_ ≤ 2 is spread slowly, and the serious epidemic when *R*_0_ ≥2 is the disease spreading very rapidly. It was found that *R*_0_ in both aggregating from the literature and our examination was >1. The output of this research alerts veterinarians, wildlife ecologists, and researchers to develop a vaccine against ASF.

However, there are significant limitations. This study only estimated with doubling time approach on a global scale; other methods, like estimating infection rate with the SIR and network modeling approaches [41, 42], could be examined and compared with model accuracy. The result may have been affected by known risk factors for ASF transmission, such as improper handling and processing of pork and pork products at slaughter slabs, butchers, and pork joints (i.e., improvised kiosks where pork is roasted and eaten), farmers' attitudes, and cultural beliefs regarding handling sick and dead animals [43]. Examination of country-wise *R*_0_ with the same data source and zonal characteristics was missing. Wild boar cases were used for the examination in this study; other host species and subspecies with age-dependent nature could be researched. The infectious period in this study was 6 days; research has reported different time periods, examining the infectious period and reevaluation with the respective period could be studied.

The disease spread and dynamics parameters such as *R*_0_, incidence rate, prevalence rate, serial interval, and contact rates are to be examined and reported frequently for sustainable preventive practice [1]. *R*_0_ is a theoretical number, so it is essential to keep in mind that it might not accurately represent how a disease spreads in practice. The actual rate of a disease's transmission can be significantly influenced by other variables, including population density, age distribution, method of transmission, the severity of the illness, the length of infectivity, the efficacy of control measures, and public health initiatives, these indexes are to be studied in the future research.

The dynamics of infectious diseases and their hosts are complex, and the impact of control programs is difficult to predict [44]. The mental model refers to a collection of assumptions that encapsulate the understanding of a complex system. “All models are wrong: but some are useful”, it is to be updated continuously [45, 46]. In this context, the proposed model focuses on a specific set of 23 variables. However, a more refined model together with control measures like surveillance, hygiene promotion, rapid response, awareness campaigns, diagnosis and treatment, data analysis, health infrastructure strengthening, advocacy for vaccination, treatment, immunization programs, wellness promotion, active community engagement, research and development, and international collaboration could enhance effectiveness. Moreover, considering geographical characteristics and host-specific behaviors in the qualitative mental model could substantially enhance its usefulness for decision-makers. Obtaining access to pertinent data related to these measures would be particularly advantageous. Such data would empower decision-makers with the necessary insights for informed and effective decision-making processes. Developing the optimized model and its scientific significance with sensitivity analysis are suggested for the following research.

In conclusion, the study deals with *R*_0_ of ASF, unveiled a concerning revelation: an *R*_0_ higher than one, indicating an alarming potential for the widespread dissemination of ASF worldwide. Further, we developed a conceptual ASFV spread and control dynamics model considering 23 variables. The insights can guide future research directions, policy formulations, and disease prevention, control, and treatment strategies. Despite significant limitations, this study can be a foundation and reference for Scholars, policymakers, and practitioners, including veterinarians, to shape more effective and comprehensive approaches for managing infectious diseases.

## Figures and Tables

**Table 1:** Reproduction number of ASF reported in different geographic regions and methods.

Host	Study area	Method	*R*_0_	Infectious period (days)	Source
Wild boar	Czech Republic	Doubling time	1.95	6	[19]
Wild boar	Belgium		1.65	6	[19]

Pig herds	Uganda	Nearest neighbor	3.24	—	[20]
		Doubling time	1.63	30	[20]
		SI-based (curve fitting, linear regression, and SI/N proportion)	(1.58, 1.90, 1.77)	—	[20]

Pig-to-pig	Georgia	SI (transmission experiment; within pen)	2.8	—	[21]
Pig-to-pig		SEIR (transmission experiment) (between pens)	1.4	—	[21]

Wild Boars	Russian	Doubling time (space–time clusters)	1.58	6	[22]
Domestic pig (between farms)		SI model	2–3	5	[23]
Domestic pig (within infected farm)		SI model	8–11	15	[23]

Domestic pig (within farm)	Ukraine	Doubling time	1.65	7	[24]
Domestic pig (between farms)		Doubling time	7.46	19	[24]

Pigs	Netherlands	Survival analysis (contact transmission experiment)	0.3	—	[18]

Pig within farm	Netherlands	Doubling time	4.92	4.6	[13]
	Malta	Doubling time	18 (6.90–46.9)	6.8	[13]
	Armenia	Doubling time	6.1 (0.6–14.5)	2–9	[25]

Wild boar	Italy	Doubling time	1.124	39	[26]
		Force of infection (*λ*)	1.165	—	[26]
		Proportion of Infected	1.17	—	[26]
		SIR model	1.139	5–7	[26]
Pig farm		Network (secondary cases)	1.86 (range 1.62 −2.82)	—	[27]

Pig farm	Vietnam	SI model	1.41–10.8	15–30	[28]
Pig herd		SEIR model	10 (1.1 to 30)	10	[29]

Wild boar	South Korea	Epidemic curves (simulation ‘who infected whom')	2.10 (range: 0.06–10.24)	—	[30]
Wild boar		Doubling time	1.01–4.38	2–9	[31]
Wild boar		Doubling time	1.54 (range: 1.11–2.37)	7.5–23.5	[8]

Pig	China	SI model	0.6	—	[32]
ASF outbreaks		—	4.83–11.90	8–11	[33]

Wild boar	Poland	Network analysis	1.1–2.5	5	[34]

SEIR: susceptible exposed infectious removed; SI: susceptible infectious; and SIR: susceptible infectious removed.

**Table 2:** Exponential growth rates and *R*_0_ in different phases.

Phase	Time frame	Days	Outbreaks	Growth rate	*R*-square	Doubling time	*R*_0_
I	01/12/2007−01/01/2014	2,224	112	0.03	0.99	26.46	1.09
II	02/01/2014−25/03/2016	814	2,498	0.43	0.94	1.60	2.52
III	26/03/2016−12/12/2017	627	1,340	0.43	0.99	1.61	2.51
IV	13/12/2017−1/12/2022	1,851	23,234	1.39	0.99	0.50	5.87
All	01/12/2007−30/12/2022	5,516	27,184	0.37	0.79	1.88	2.29

## Data Availability

Anonymized data will be made available by the corresponding author upon request.
